# Systematic sequence analysis of the *FUT3* gene identifies 11 novel alleles in the Sindhi and Punjabi populations from Pakistan

**DOI:** 10.1038/s41598-020-62524-8

**Published:** 2020-03-26

**Authors:** Maomao Zhao, Atif Adnan, Allah Rakha, Shahid Nazir, Meihui Tian, Siyi Zhang, Hao Pang

**Affiliations:** 1 School of Forensic Medicine, Department of Forensic Genetics and Biology, China Medical University, Shenyang, 110122 China; 2 Department of Forensic Sciences, University of Health Sciences, Lahore, 54600 Pakistan

**Keywords:** Rare variants, Evolutionary biology

## Abstract

The *FUT3* (*Lewis*) gene is responsible for the expression of Lewis fucosyltransferase, which is required for the synthesis of the structural determinants of both Lewis^a^ and Lewis^b^ specificity. These factors play an important role not only in clinical but also in medico-legal investigations. The gene sequence is highly polymorphic and ethnically specific. In the current study, we performed systematic sequence analysis of the coding region of *FUT3* by DNA sequencing to investigate the genetic variations of *FUT3* and the molecular basis of the Lewis phenotype in the Sindhi and Punjabi populations of Pakistan. Twenty-three point mutations were observed, including 7 unreported mutations, among which two missense mutations (490 G > A and 959 T > C) were predicted to be deleterious to enzyme activity by software assessment. In total, we observed 24 Lewis alleles, including 11 novel ones. However, all unreported missense mutations were present in Lewis-negative alleles confirmed previously. According to genotypic data, the Lewis-negative phenotypic frequencies were 11.5% and 22.93% in the Sindhi and Punjabi ethnic groups, respectively. Moreover, we found that *le*^*202,314*^ and *le*^59*,1067*^ were predominant among Lewis-negative alleles, while the frequency of *le*^*59,1067*^ in the Punjabi population was significantly higher than that in the Sindhi population. In summary, our study revealed that there is a relatively high degree of sequence variation of the *Lewis* gene in Pakistani populations and provided the first genetic data on *FUT3* in these two ethnic groups from Pakistan. The allele types and their frequencies showed that these ethnic groups exhibit more Caucasian components.

## Introduction

 α (1,3/1,4) FucT (fucosyltransferase), encoded by the *FUT3* (*Lewis*) gene, regulates the expression of Lewis antigens in the human Lewis blood group system. The human Lewis blood group system is mainly composed of two Lewis antigens, Le^a^ and Le^b^. These were initially identified as red blood cell (RBC) antigens, but were later discovered in exocrine secretions that were not inherent to RBCs but were absorbed against erythrocyte membranes from plasma^1^. In this context, there are some complications in regard to the phenotyping of the Lewis system using the haemagglutination test due to poor antibody specificity^2^. Other phenotyping errors with physiological and pathological causes include the following: (1) some individuals whose erythrocytes are typed as Lewis positive on can subsequently show a Lewis-negative erythrocyte phenotype during disease or pregnancy^3^; (2) because of the gene dosage effect causing *FUT3* heterozygous individuals to exhibit lower α1-4 FucT activity in secretions than homozygous wild-type individuals, genuine Lewis-positive individuals (Le (functional)/le (nonfunctional) may erroneously be typed as showing a Lewis-negative phenotype on erythrocytes^4^; (3) the histo-blood group A-glycosyltransferase, B-glycosyltransferase and Lewis fucosyltransferase act on a common precursor substance, H type-1, and this competition will result in a reduction of the Le^b^ antigen in A and B individuals compared to individuals with blood group O. Thus, some Le^b^ antibodies may falsely type Le(a-b+) as Le(a-b−)^5^. In addition, if we want to acquire phenotype information on the Lewis blood system from biological materials other than whole blood, such as bloodstains (which we used in this study), body fluid, or hair, we may need to determine the genotype of the Lewis blood group at the DNA level to infer the corresponding phenotype. Therefore, it is important to determine the genotypes of the Lewis system.

Despite being relatively uncommon, in contrast to the ABO and RH systems, the Lewis blood type displays clinical significance. A few examples of haemolytic transfusion reactions have been attributed to improper phenotyping by using Lewis antibodies^6,7^. Lewis antibodies have also been implicated in mild symptoms of HDN because Lewis phenotypes might be falsely typed in red cells from women and infants^8^. In addition, the identical donor–recipient pairs based on Lewis phenotypes were shown to have better graft survival than Lewis-incompatible pairs^9^. Therefore, it is necessary for the Lewis blood group system to be appropriately considered in transfusion and transplantation.

The protein structure of the Lewis enzyme is composed of an NH_2_-terminal cytoplasmic tail, a transmembrane region, a stem region and a COOH-terminal catalytic domain^10^. The point mutations in the coding sequence of *FUT3* affecting Lewis enzyme activity depend on the corresponding amino acid characteristics and protein structure. The mutations like T202C, G508A, G667A, G808A, A1007C, T1067A will completely lose enzyme activity, while also some mutations like T59G, G484, C478T, G968C will lead to portion loss of it^1,11–13^. The G47C and G1022T mutations are also predicted to inactivate the enzyme^14,15^. The alleles composed of inactivating mutations are referred to as Lewis-negative alleles (*le*). An individual homozygous for *le* loses the ability to express the Le^a^ and Le^b^ antigens and presents the Lewis-negative phenotype Le (a-b-) on the red blood cell membrane. The alleles with mutations that do not influence enzyme activity are referred to as Lewis-positive alleles (*Le*).

The most common and important Lewis-negative alleles are *le*^*202,314*^, *le*^*59,1067*^, *le*^*59,508*^, *le*^*484,667*^, and other rare Lewis-negative alleles originating from them^13^. Moreover, these alleles show racial differences and specificity. For instance, *le*^*202,314*^ and *le*^*59,1067*^ are found mainly in European populations, while *le*^*59,508*^ is common in east Asian and African populations^13,16^. To date, *le*^*484,667*^ has been detected only in African populations^1,13^. Previous studies have focused on Lewis-negative genes in many countries in Asia, including East Asian countries such as China^2^, Japan^17^, Korea^18^, and Mongolia^13^; Southeast Asian countries such as the Philippines^11^, Thailand^11^, and Indonesia^19^; and South Asian countries such as Sri Lanka^14^. However, there are no reported genetic data on *FUT3* from Pakistan, which is a multi-ethnic country located in South Asia. Thus, in this study, we performed a systematic sequence analysis of the *Lewis* gene coding region by sequencing to investigate the genetic variations of *FUT3* and the molecular basis of the Lewis phenotype in Sindhi and Punjabi populations from Pakistan and to better understand the genetic origin of the Pakistani population, in combination with other reports about ancestry-informative markers.

## Results

In the current study, the distribution of *FUT3* alleles was in Hardy-Weinberg equilibrium. Here, we defined mutations with an rs number in the dbSNP database (https://www.ncbi.nlm.nih.gov/projects/SNP/snp_ref.cgi?geneId=2525) and defined mutations without population data from published reports and in the 1000 Genomes browser (https://www.ncbi.nlm.nih.gov/variation/tools/1000genomes/?assm=GCF_000001405.25) as unreported mutations.

### Sequence variations in the coding region of *FUT3*

We found 18 single nucleotide polymorphisms (SNPs) in the Sindhi population and 12 in the Punjabi population of Pakistan as a result of DNA sequencing from the whole coding region of the *FUT3* locus. Among the 18 SNPs found in the Sindhi population, 14 were reported previously, while 4 were unreported, among which G146A (rs1263565737) and G490A (rs767305253) were missense mutations, and G381A (rs144354196) and G561A (rs747036561) were synonymous mutations. Moreover, in the Punjabi population, in addition to 9 previously reported mutations, we observed 3 unreported mutations, among which 2 were synonymous mutations (G24C (new mutation submitted to dbSNP database) and C876T (rs3934140)), and 1 was a missense mutation (T959C (rs762649552)). All SNPs identified in this study and their corresponding frequencies are summarized in Table 1.

**Table 1:** All mutations identified in *FUT3* coding region from Sindhi and Punjabi populations.

	Sindh				Punjabi			
	Number	SNP	rs number	Frequency	Number	SNP	rs number	Frequency
Unreported	1	G146A	rs1263565737	1 (0.25%)	1	G24C	New mutation	1 (0.32%)
	2	G381A	rs144354196	1 (0.25%)	2	C876T	rs773934140	1 (0.32%)
	3	G490A	rs767305253	1 (0.25%)	3	T959C	rs762649552	1 (0.32%)
	4	G561A	rs747436561	1 (0.25%)				
Reported	5	T59G	rs28362459	68 (17.00%)	4	T59G	rs28362459	75 (23.89%)
	6	T202C	rs812936	87 (21.75%)	5	T202C	rs812936	68 (21.66%)
	7	C314T	rs778986	81 (20.25%)	6	C314T	rs778986	62 (19.75%)
	8	G508A	rs3745635	15 (3.75%)	7	G508A	rs3745635	12 (3.82%)
	9	T1067A	rs3894326	57 (14.25%)	8	T1067A	rs3894326	59 (18.79%)
	10	G47C	rs145362171	9 (2.25%)	9	G47C	rs145362171	15 (4.78%)
	11	T645C	rs148170391	6 (1.50%)	10	C882T	rs778985	2 (0.64%)
	12	A612G	rs28362465	4 (1%)	11	C445A	rs143012663	1 (0.32%)
	13	G13A	rs28362458	1 (0.25%)	12	T645C	rs148170391	1 (0.32%)
	14	G484A	rs28362463	1 (0.25%)				
	15	G667A	rs28362466	1 (0.25%)				
	16	A1007C	rs151218854	1 (0.25%)				
	17	G113A	rs147046153	1 (0.25%)				
	18	G1061A	rs144953440	1 (0.25%)				

### Novel haplotypes and inference of the influence of new missense mutations on enzyme activity

We identified the *Lewis* haplotypes of 11 individuals who carried unreported mutations and undefined haplotypes via PCR cloning and allele-specific PCR. Finally, we identified eleven novel alleles, among which seven were defined by seven unreported mutations, one resulted in a new combination involving the previously reported mutation C882T, and the remaining three were characterized by the presence of G113A, G1061A, and T645C, which have been reported in the 1000 Genomes database, but haplotypes consisting of these three mutations are still unknown. Among these eleven alleles, seven were non-functional, and four were functional, which are summarized in Table 2. Hence, all nonsynonymous mutations found in this study were included in the Lewis-negative alleles confirmed previously, such as the G146A and G1061A mutations located on the chromosome of *le*^*59,508*^; G490A and T959C located on the chromosome of *le*^*59,1067*^; and G113A located on *le*^*202,314*^. Under these conditions, the Lewis enzyme activities generated from the non-functional alleles with these mutations were not examined further. However, we can speculate about the impact of these nonsynonymous substitutions on enzyme activity on the basis of the positions of mutations and the associated changes in amino acids using the PolyPhen and PROVEAN programs. The G113A (R38Q) and G146A (S49N) mutations lie in the stem region of the encoded protein structure, showing a relatively minor effect on enzyme activity, and both were predicted to be benign (score: G113A:0.002; G146A:0) by PolyPhen and to be neutral by PROVEAN. The other three missense mutations, G490A (D164N), T959C (F320S) and G1061A (R354H), are located in the catalytic domain of the enzyme and are more likely to influence enzyme activity. However, the G1061A mutation was predicted to be benign (score: 0) by PolyPhen and to be neutral by PROVEAN, possibly because this mutation does not change the biochemical properties of the amino acids (arginine and histidine are both alkaline amino acids). The G490A (D164N) and T959C (F320S) mutations are predicted to be damaging (score: G490A: 1.0; T959C: 0.989) by PolyPhen and lethal (score: G490A: −4.878; T959C: −7.255) by PROVEAN. A mutation with a score ≤ −2.5 is considered lethal, while a score > −2.5 indicates a neutral mutation.

**Table 2:** New haplotypes and genotypes identified in *FUT3* gene in Sindhi and Punjabi populations.

Population	Sample ID	Mutations	Other mutations	Primer used by SSP	Genotype	New haplotype
Sindhi	F6	G381A*	T202C; C314T	201G-202C-U/Nest-FUT3-L	*le*^*202,314*^*/Le*^*381*^	*Le*^*381*^
	37	G561A*	—	—	*Le/Le*^*561*^	*Le*^*561*^
	K9	T645C^#^	T202C; C314T	201G-202C-U/Nest-FUT3-L	*Le*^*645*^*/le*^*202,314*^	*Le*^*645*^
	K6	G146A*	T59G; G508A	59G-U/Nest-FUT3-L	*Le/le*^*59,146,508*^	*le*^*59,146,508*^
	97	G113A^#^	T202C; C314T	113A-U/Nest-FUT3-L	*Le/le*^*202,314*^	*le*^*113,202,314*^
	C9	G1061A^#^	T59G; G508A	59G-U/Nest-FUT3-L	*Le/le*^*59,508,1061*^	*le*^*59,508,1061*^
	36	G490A*	G47C; T59G; T202C	59G-U/Nest-FUT3-L	*le*^*47,202,314*^*/le*^*59,490,1067*^	*le*^*59,490,1067*^
			C314T; T1067A			
Punjabi	M61	C876T*	—	—	*Le/Le*^*876*^	*Le*^*876*^
	F38	G24C*	T59G; G508A	23C-24C-U/Nest-FUT3-L	*Le/le*^*24,59,508*^	*le*^*24,59,508*^
	F29	C882T*	T202C; C314T	201G-202C-U/Nest-FUT3-L	*Le/le*^*202,314,882*^	*le*^*202,314,882*^
	F45	T959C*	T59G; T1067A	59G-U/Nest-FUT3-L	*Le/le*^*59,959*,1067^	*le*^*59,959*,1067^

*Unreported mutation; #reported mutation.

*Le*: Lewis-positive allele; *le*: Lewis-negative allele.

### Phenotype, genotype, and allele frequencies

As shown in Table 3, we identified 18 and 14 alleles and 29 and 24 genotypes in the Sindhi and Punjabi populations, respectively. The seven alleles *Le*, *Le*^*59*^, *le*^*59,1067*^, *le*^*202,314*^, *le*^*202,314,47*^, *le*^*59,202,1067*^, *le*^*59,508*,^ and *Le*^*645*^ were found in both the Sindhi and Punjabi populations, whereas the *le*^*1007*^, *le*^*1067*^, *le*^*13,484,667*^, and *Le*^*612*^ alleles were only present in the Sindhi population, and *le*^202^ and *le*^*59,445*^ were only found in the Punjabi population (novel alleles are shown in Table 2). Notably, the three most common *FUT3* alleles in the two populations were *Le*, *le*^*59,1067*^ and *le*^*202,314*^, and the frequency of these alleles in the Sindhi population was 87%, while it was 87.57% in the Punjabi population. Non-functional *Lewis* alleles accounted for 38.5% of the alleles in the Sindhi population, among which the *le*^*202,314*^ was the most common (17.75%). In the Punjabi population, 43.63% of alleles were non-functional, and *le*^*59,1067*^ was the most frequent (17.52%), showed significant differences between the two populations (*P* < 0.05). Moreover, the 3 alleles *le*^*47,202,314*^, *le*^*59,202,1067*^ and *le*^*202*^ were relatively rare, but *le*^*47,202,314*^ (2.25% in Sindhi and 4.78% in Punjabi) and *le*^*59,202,1067*^ (1.25% in the Sindhi and 1.27% in the Punjabi populations) presented a considerable frequency in these studied populations. According to the genotypic data, the frequency of the Lewis-negative phenotype was 11.5% in the Sindhi population and 22.93% in the Punjabi population, indicating a higher frequency of the Lewis-negative phenotype in the Punjabi population.

**Table 3:** Frequencies of allele, genotype, and phenotype in *FUT3* locus from Sindhi and Punjabi populations.

Sindh		Punjabi		Sindhi			Punjabi		
Allele	No (%)	Allele	No (%)	Phenotype	Genotype	No (%)	Phenotype	Genotype	No (%)
**Nonfunctional**		**Nonfunctional**		*le* (11.5%)	*le*^*202,314*^*/le*^*202,314*^	5 (2.5)	*le* (22.93%)	*le*^*202,314*^*/le*^*202,314*^	8 (5.1)
**Total**	154 (38.5)	**Total**	137 (43.63)		*le*^*202,314*^*/le*^*59,1067*^	4 (2)		*le*^*202,314*^*/le*^*59,1067*^	9 (5.73)
*le*^*202,314*^	71 (17.75)	*le*^*202,314*^	46 (14.65)		*le*^*47,202,314*^*/le*^*202,314*^	2 (1)		*le*^*47,202,314*^*/le*^*202,314*^	3 (1.91)
*le*^*47,202,314*^	9 (2.25)	*le*^*47,202,314*^	15 (4.78)		*le*^*59,1067*^*/le*^*59,1067*^	4 (2)		*le*^*59,1067*^*/le*^*59,1067*^	6 (3.82)
*le*^*59,1067*^	45 (11.25)	*le*^*59,1067*^	55 (17.52)		*le*^*59,1067*^*/le*^*59,202,1067*^	2 (1)		*le*^*59,1067*^*/le*^*59,202,1067*^	1 (0.64)
*le*^*59,202,1067*^	5 (1.25)	*le*^*59,202,1067*^	4 (1.27)		*le*^*59,508*^*/le*^*59,1067*^	3 (1.5)		*le*^*59,508*^*/le*^*59,1067*^	4 (2.55)
*le*^*59,508*^	12 (3.00)	*le*^*59,508*^	11 (3.50)		*le*^*1067*^*/le*^*1067*^	1 (0.5)		*le*^*202,314*^*/le*^*202,314,882*^	1 (0.64)
*le*^1007^	1 (0.25)	*le*^*202*^	1 (0.32)		*le*^*13,484,667*^*/le*^*59,202*,1067^	1 (0.5)		*le*^*47,202,314*^*/le*^*47,202,314*^	1 (0.64)
*le*^*1067*^	6 (1.5)	*le*^*59,445*^	1 (0.32)		*le*^*47,202,314*^*/le*^*59,490,1067*^	1 (0.5)		*le*^*47,202,314*^*/le*^*59,1067*^	2 (1.27)
*le*^*13,484,667*^	1 (0.25)	*le*^*24,59,508*^	1 (0.32)					*le*^*47,202,314*^*/le*^*59,202,1067*^	1 (0.64)
*le*^*113,202,314*^	1 (0.25)	*le*^*202,314,882*^	2 (0.64)	*Le* (88.5%)	*Le/Le*	64 (32)	*Le* (77.07%)	*Le/Le*	53 (33.76)
*le*^*59,146,508*^	1 (0.25)	*le*^*59,959,1067*^	1 (0.32)		*Le/le*^*202,314*^	50 (25)		*Le/le*^*202,314*^	17 (10.83)
*le*^*59,490,1067*^	1 (0.25)				*Le/le*^*47,202,314*^	6 (3)		*Le/le*^*47,202,314*^	7 (4.46)
*le*^*59,508,1061*^	1 (0.25)				*Le/le*^*59,1067*^	28 (14)		*Le/le*^*59,1067*^	27 (17.20)
**Functional**		**Functional**			*Le/le*^*59,202,1067*^	3 (1.5%)		*Le/le*^*59,202,1067*^	2 (1.27)
**Total**	246 (61.5)	**Total**	177 (56.37)		*Le/le*^*59,508*^	6 (3)		*Le/le*^*59,508*^	7 (4.46)
*Le*	232 (58)	*Le*	174 (55.41)		*Le/Le*^645^	2 (1)		*Le/Le*^645^	1 (0.64)
*Le*^59^	2 (0.50)	*Le*^59^	1 (0.32)		*Le*^59^*/le*^202*,314*^	1 (0.5)		*Le/le*^*202*^	1 (0.64)
*Le*^612^	4 (1.00)	*Le*^645^	1 (0.32)		*Le*^59^*/Le*^612^	1 (0.5)		*Le/le*^*202,314,882*^	1 (0.64)
*Le*^645^	6 (1.50)	*Le*^876^	1 (0.32)		*Le*^612^*/le*^*202,314*^	2 (1)		*Le/le*^*24,59,508*^	1 (0.64)
*Le*^381^	1 (0.25)				*Le*^645^*/le*^*202,314*^	1 (0.5)		*Le/Le*^59^	1 (0.64)
*Le*^561^	1 (0.25)				*Le*^645^*/le*^*59,508*^	3 (1.5)		*Le/le*^*59,445*^	1 (0.64)
					*Le/le*^*113,202,314*^	1 (0.5)		*Le/le*^*59,959,1067*^	1 (0.64)
					*Le/le*^1007^	1 (0.5)		*Le/Le*^876^	1 (0.64)
					*Le/le*^1067^	3 (1.5)			
					*Le/Le*^561^	1 (0.5)			
					*Le/le*^*59,146,508*^	1 (0.5)			
					*Le/le*^*59,508,1061*^	1 (0.5)			
					*Le/Le*^612^	1 (0.5)			
					*Le*^381^*/le*^*202,314*^	1 (0.5)			

## Discussion

Pakistan lies in a region that has been invaded by several different groups in the past, including Greeks, Aryans, Macedonians, Arabs and Mongols^20^. These invaders contributed to the ethnic variety of the Pakistani populations. There are many ethnic groups inhabiting different parts of Pakistan. In this study, the systematic sequencing analysis of the coding region of the *FUT3* gene was performed in two ethnic groups, Punjabis, representing 62% of the Pakistani population, and Sindhis representing 18%. In the context of the Lewis blood type and genetic polymorphism mentioned above, these two groups were appropriate for in the current study because they represent>78% of the total Pakistani population. The Lewis blood group system is not only highly polymorphic but also ethnically and geographically specific. Many different sequence variations have been observed in different populations around the world. According to our results, the studied populations exhibit higher sequential variation and a wide variety of alleles at the *FUT3* locus.

Initially, the most frequent mutations identified in the studied populations were T59G, T202C, C314T, G508A, T1067A and G47C (as shown in Table 1). The T59G mutation was either present as a singleton or linked with other mutations such as G508A and T1067A. The G508A mutation is most commonly found in Asian, African and Amazonian populations^1,13,21,22^, but in the currently studied populations, the frequency of this mutation only accounts for 3.75% (Sindhi) and 3.82% (Punjabi). On the other hand, the T1067A mutation, which is frequent in Japanese^17^, Sinhalese^14^, Southeast Asian^11^, and Caucasian populations^13^, also represents 14.25% in Sindhis and 18.79% in Punjabis. The T202C and C314T mutations, which were found predominantly in the Sindhi (21.75% and 20.25%) and Punjabi (21.66% and 19.75%) populations, have most commonly been found in Caucasian populations^13^. In most cases, T202C and C314T are in complete linkage, but it was interesting that the T202C singleton, which has been previously identified in Xhosa and Caucasian populations^1^, was also found in a heterozygous individual from the Punjabi population. It is worth noting that the G47C mutation, which has only previously been identified in the Sinhalese population of Sri Lanka in South Asia^14^ and the Caucasian panel of Coriell Cell Repositories, was found at a notable frequency in the Sindhi (2.25%) and Punjabi (4.78%) populations, indicating that G47C of *FUT3* may be more specifically present in South Asian populations. In addition, some other rare mutations were sporadically identified in the investigated populations. For example, the G13A mutation, which was originally found in African Americans and was common in native Africans, was also present in the Sindhi population^1,15^. C445A was originally observed in Denmark^4^ and A1007C has only been reported in Japanese populations^12^; both enzyme-inactivating mutations were also seen in one heterozygous individual in each of the Punjabi and Sindhi populations. The A612G mutation was found only in Mongols and was identified in 4 heterozygous individuals from the Sindhi population^13^, while the T645C mutation was shared by both the Sindhi and Punjabi populations. The distribution characteristics of common and rare mutations at the *Lewis* locus suggested the existence of extensive sequence diversity in the *Lewis* coding region in the Sindhi and Punjabi populations, and the *FUT3* SNPs and alleles shared with other racial populations indicated a mixed trait in the two investigated populations of Pakistan.

Second, more alleles of the human Lewis blood group, including seven novel non-functional alleles and four functional alleles, were found in the current study. Interestingly, all novel non-functional alleles came from the known Lewis-negative alleles with additional mutations. Previously, 90–95% Lewis-negative individuals were identified in Caucasians by screening the four SNPs, T59G, T1067A, T202C, and C314T^23,24^. Our results showed that the addition of G508A to the above four SNPs was sufficient to define the Lewis-negative alleles in Pakistani populations.

According to the frequency distribution of the Lewis allele and the negative phenotype in the Sindhi and Punjabi populations, the frequency of *le*^*59,1067*^ in the Punjabi population was significantly higher than that in the Sindhi population, although the statistical analysis indicated that the whole frequency distribution of alleles showed no significant differences (*P* > 0.05). Furthermore, the Lewis-negative phenotype frequency of the Punjabi population (22.93%) was twice that of the Sindhi population (12%). Therefore, the genetic profile of the Lewis blood type system in these two groups is somewhat similar.

The type and frequency distribution of the Lewis alleles, especially non-functional Lewis alleles, are race-specific among many populations^1,13,14,17,18,21,22^ (Table 4). *le*^*59,508*^ is commonly found in East Asian and African populations, but the frequency is relatively lower in the currently investigated populations. *le*^*202,314*^ is commonly found in Caucasians and Sinhalese ethnic groups of South Asia. Importantly, the highest frequency of *le*^*59,1067*^ was observed in the Sinhalese, followed by the Caucasians and Japanese populations. The *le*^*202,314*^ and *le*^*59,1067*^ alleles are mainly non-functional alleles in the Punjabi and Sindhi populations. Moreover, *le*^*47,202,314*^ and *le*^*59,202,1067*^ are rarely observed in other populations but were frequently found in currently studied populations. A study addressing mitochondrial control region diversity in the Sindhi population showed that the haplogroups constituting the mtDNA library were mainly derived from South Asia (47.6%) and West Eurasia (35.7%)^20^. Likewise, the Punjabi mtDNA gene pool is primarily a composite of considerable proportions of South Asian haplotypes (65%) and West Eurasian (29%) haplogroups^25^. Therefore, based on the distribution of alleles at the *FUT3* locus, we can conclude that the Punjabi and Sindhi populations from Pakistan are more closely related to Sinhalese and Caucasian population, and the present results conform to those of many other studies in Sindhi and Punjabi populations.

**Table 4:** Comparison of allele frequencies of *FUT3* gene among different populations.

Alleles	Sindhi	Punjabi	Sinhalese^14^	Chinese^22^	Japanese^17^	Mongolians^13^	Korean^18^	Ghanaians^13^	Xhosa^1^	Caucasians^13^	Amazonian^21^
Total (chromosomes)	400	314	108	632	298	100	484	212	200	200	300
*Le*	57.75%	55.41%	52.80%	72.94%	60.70%	56.00%	73.10%	42.00%	50.00%	70.50%	49.70%
*Le*^59^	0.50%	0.32%	nd	4.75%	0.50%	nd	1.00%	nd	nd	nd	29.00%
*other Le*	3.00%	0.64%	nd	nd	—	5.00%	—	2.40%	2.50%	nd	—
*le*^1067^	1.25%	nd	nd	0.32%	0.30%	nd	nd	nd	nd	nd	2.00%
*le*^202*,314*^	17.75%	14.65%	12.00%	2.85%	nd	5.00%	—	6.60%	8.00%	17.00%	0.70%
*le*^*59,508*^	3.00%	3.50%	1.90%	14.72%	27.50%	24.00%	22.30%	18.90%	31.00%	1.50%	15.30%
*le*^*59,1067*^	11.25%	17.52%	28.70%	4.27%	11.40%	3.00%	3.50%	1.40%	2.50%	13.00%	2.00%
*le*^*47,202,314*^	2.25%	4.78%	2.80%	nd	—	nd	nd	nd	nd	2.50%	nd
*le*^*59,202,1067*^	1.25%	1.27%	1.90%	nd	nd	nd	nd	nd	nd	1.00%	nd
*le*^*202*^	nd	0.32%	nd	nd	—	nd	—	nd	1.50%	1.00%	—

nd: not detected; -: not examined.

In recent years, new interest in the polymorphisms of *FUT3* has been raised by genome-wide association studies (GWASs), which have suggested inactivating polymorphisms (T59G, G508A) of the gene to be associated with the prevalence of ulcerative colitis (UC)^26^, Crohn’s disease (CD)^27^ and coronary artery disease^28,29^. Moreover, these two SNPs also influence the lesion location in UC and CD. In the Sindhi and Punjabi populations, our results demonstrated that T59G, G508A, T1067A, T202C, and C314T, as tag SNPs of Lewis-negative alleles, will be useful for large-scale association studies of Lewis-negative phenotypes with diseases in the future.

On the other hand, recent studies have suggested that the Lewis phenotype is associated with susceptibility to infection by *Norovirus*^30^, *Rotavirus*^31^ and *Helicobacter pylori*^32^. The Lewis-negative phenotype is resistant to norovirus (GI) and rotavirus (P8), which are the leading causes of acute gastroenteritis in children worldwide. According to a previous report, the Lewis-negative phenotype varies from 7% (Asians) or 8% (Europeans) to 19% (Africans)^21^. Our results showed that the frequencies of the Lewis-negative phenotype were 12% (Sindhi) and 22.93% (Punjabi). Thus, a stable proportion of the Lewis-negative phenotype is maintained under long-term natural selection. The reason may be associated with microorganism infection, possibly related to a protective strategy against widespread disease.

In the past, it was relatively difficult to accurately classify Lewis phenotypes using the haemagglutination test in medico-legal investigations. This method only works for whole blood samples, not for special materials such as body fluid, hair, and bloodstains that are commonly found at crime scenes. However, in this study, we successfully typed the Lewis phenotype using bloodstain samples.

In conclusion, multiple sequence variations and a wide variety of alleles, including eleven novel alleles of *FUT3*, were identified by systematic sequencing analysis in Punjabi and Sindhi populations. These populations were not previously studied in reference to *FUT3*, and our present study revealed that the Sindhi and Punjabi populations are a mixture of South Asian and Caucasian ancestry. Thus, a genetically better understanding of the origins of these two ethnic groups is presented in this research.

## Materials and Methods

### Sample collection

In the current study, we collected bloodstains on FTA cards from 357 (200 Sindhi and 157 Punjabi) unrelated individuals residing in Sindh and Punjab provinces of Pakistan. All participants gave their informed consent either orally and with a thumb print (if they could not write) or in writing after the study aims and procedures were carefully explained to them. The study was approved (2019/060) by the ethical review board of China Medical University, Shenyang, Liaoning Province, People’s Republic of China, and was performed in accordance with the standards of the Declaration of Helsinki.

### DNA isolation

Genomic DNA was isolated from FTA bloodstain cards using a modified phenol-chloroform method developed by our group (Supplemental File 1). The extracted DNA samples were quantified using a NanoDrop spectrophotometer (Thermo Scientific, Wilmington DE, USA).

### PCR amplification of the *FUT*3 genes

A DNA fragment (1226 bp) containing the open reading frame (1086 bp) was first amplified by PCR in a 20 μl system including 10 μl of 2×Power Taq PCR MasterMix (Bioteke, Beijing, China), 40 ng genomic DNA, and 5 μmol of each primer. The primer sequences are shown in Table 5. PCR was carried out under following conditions: initial denaturing at 94 °C for 5 min, followed by 35 cycles of denaturing at 94 °C for 30 s, annealing at 65 °C for 30 s, and extension at 72 °C for 1 min.

**Table 5:** Sequences and positions of PCR primers and annealing condition used for analysis of the Lewis gene.

Primers	Sequences (5′ → 3′)	Positions	Annealing temperature
FUT3-U	GCAGCTCCTCTCAGGACTCATGGCCC	−100~−75	65 °C
FUT3-L	CAGATGAGGTTCCCGGCAGCCCAGGCA	1100~1126	
Nest-FUT3-U	CCCAAGCTTCACTCCTCTCTCCTCTCTT	−36~−18	62.4°C
Nest-FUT3-L	TGCTCTAGACAGATGAGGTTCCCGGCAGCC	1106~1126	
59G-U	CGCCGCTGTCTGGCCGCACG	40~59	68.6°C
201G-202C-U	CACCCTCCTGATCCTGCTGC	183~202	62.4°C
113A-U	TCCTACCTGCGTGTGTCCCA	94~113	65°C
23C-24C-U	ATCCCCTGGGTGCAGCCACC	5~24	60°C
Seq-U	GCAGCGACTCCGACATCTTC	479~498	
Seq-L	GTAGCGCACCCTGGCTGAGT	608~627	

### Direct DNA sequencing

The synthesized PCR products were directly sequenced using Sanger sequencing with the sequencing primers (shown in Table 5). The sequencing conditions were described previously^33^.

### Haplotype identification

Nested PCR was carried out using a 20 μl system containing 10 μl of 2× Power Taq PCR MasterMix (Bioteke, Beijing, China), 2 μl of the 1000-fold-diluted first PCR product and 5 μmol of each primer in 11 individuals, which showed unreported and rare point mutations. The primer sequences are shown in Table 5. The PCR conditions were the same as for the first round of PCR. The obtained PCR products were digested by the restriction enzymes *Hin*d III and *Xba* I. These target regions were then subcloned into pcDNA3.1. For the determination of individual haplotypes, a minimum of four clones of each plasmid were sequenced.

To verify our results for individual haplotypes, we also performed allele-specific PCR in a 20 μl system containing 2 μl of 10× PCR buffer, 1 μl dNTP mix, 5 μmol of each primer, 2.5 units rTaq DNA polymerase and the 1000×-diluted first PCR products as templates. We designed a total of 4 upstream primers and a common downstream primer (Nest-FUT3) for amplification in all individuals. The primer sequences are shown in Table 5. The PCR conditions were as follows: 94 °C initial denaturation step for 5 min, followed by 25 cycles of 30 s at 94 °C, 30 s at the annealing temperature (Table 5), and 1 min at 72 °C. The allele-specific PCR products were sequenced by the Sanger sequencing method.

### Statistical analysis

The DNA sequences were analysed by using DNAMAN8 software with the NCBI sequence NG_007482 as a reference. Allelic frequencies and genotypes were calculated by the direct counting method, while Hardy-Weinberg equilibrium (HWE) was assessed with the chi-square test. The differences in the allele frequency distribution between the currently studied populations and reference populations were calculated by using SPSS version 21.0 software. The effect of point mutations on enzyme activity was inferred with the PolyPhen and PROVEAN programs.

## Supplementary information

Supplementary information
