# Supplementary material for: Systematic sequence analysis of the FUT3 gene identifies 11 novel alleles in the Sindhi and Punjabi populations from Pakistan
Source: Sci Rep. 2020 Mar 26;10:5543. doi: 10.1038/s41598-020-62524-8 (PMC7099025; doi:10.1038/s41598-020-62524-8)
Supplement: Supplementary file 1 — Supplementary information [file 41598_2020_62524_MOESM1_ESM.docx]

**SUPPLEMENTARY FILE 1
Protocols to extract genome DNA from FTA bloodstain card**

1. **Cut the FTA bloodstain card (1 cm^2^) into pieces and place it in an** **1.5 mL microtube, add an appropriate amount of distilled water (300 μl) at 56℃ to soak the bloodstain fragments, and put the 1.5 mL microtube into a water bath at 56℃ for heat preservation. The purpose of this procedure is to dissolve the blood stain sufficiently.**
2. Add the lysis buffer, the tubes should not be more than one-third full.
3. Add proteinase K (20 mg/ml) to a final concentration of 100 μg/ml.
4. Incubate the lysate in a water bath for 3 hours at 56℃ or overnight at 37℃. Gently mix the lysates manually from time to time.
5. Cool the solution to room temperature and add an equal volume of phenol equilibrated with 0.1 M Tris-Cl (pH 8.0). Gently mix the two phases by slowly turning the tube end-over-end for 10 minutes on a tube mixer or roller apparatus. If the two phases have not formed an emulsion at this stage, place the tube on a roller apparatus for 1 hour.
6. Separate the two phases by centrifugation at 5000 g for 15 minutes at room temperature.
7. **Use a pipette to gently transfer the viscous aqueous phase to a fresh 1.5 mL microtube. Note that the FTA card is not removed until this step.**
8. Add equal volume of phenol chloroform mixture (1:1), and then mix the two phases by slowly turning the tube end-over-end for 10 minutes on roller apparatus, finally separate the two phases by centrifugation at 5000 g for 15 minutes at room temperature.
9. Transfer the aqueous phase to a fresh 1.5 mL microtube. Add equal volume of chloroform and the rest steps are the same as above.
10. **Transfer the aqueous phase to a fresh 1.5 mL microtube, then add 1/10 volume of sodium acetate (3 mol/L) and 2 volumes of ice ethanol and swirl the tube until the solution is thoroughly mixed, finally put the solution in the -20℃ freezer for overnight to further precipitate the DNA, which can greatly increase the production of DNA.**
11. Collect the precipitate by centrifugation at 10000 g for 15 minutes at 4℃.
12. Wash the precipitate twice with 70% ethanol, and collect the DNA by centrifugation as described in step 11.
13. Remove as much of the 70% ethanol as possible, using an aspirator. Store the pellet of DNA in an open tube at room temperature until the last visible traces of ethanol have evaporated.
14. Add TE to dissolve DNA and store it.

Annotation: Font bold steps are different from protocols of chapter 6-1 of Molecular Cloning: A Laboratory Manual (the condensed protocols)
